# Identifying priorities for balance interventions through a participatory co-design approach with end-users

**DOI:** 10.1186/s12883-023-03312-5

**Published:** 2023-07-13

**Authors:** Natasha L. Benn, Hope Jervis-Rademeyer, Kayla Benson, Katherine Chan, Jae W. Lee, Elizabeth L. Inness, Dalton L. Wolfe, Milad Alizadeh-Meghrazi, Kei Masani, Kristin E. Musselman

**Affiliations:** 1 Rehabilitation Sciences Institute, University of Toronto, Toronto, Canada; 2 KITE Research Institute-Toronto Rehabilitation Institute, University Health Network, Toronto, Canada; 3 Faculty of Medicine, University of Alberta, Edmonton, Canada; 4 Institute of Biomedical Engineering, University of Toronto, Toronto, Canada; 5 Parkwood Institute, Lawson Health Research Institute, London, Canada; 6 Myant Inc, Etobicoke, Canada; 7 Department of Physical Therapy, University of Toronto, Toronto, Canada; 8 SCI Mobility Lab, KITE-Toronto Rehabilitation Institute, UHN, 520 Sutherland Drive, Toronto, M4G 3V9 Canada

**Keywords:** Functional electrical stimulation, Balance control, Rehabilitation, Stroke, Spinal cord injury, Healthcare professionals, Qualitative research, Participatory research

## Abstract

**Background:**

Most individuals living with spinal cord injuries/diseases (SCI/D) or stroke experience at least one fall each year; hence, the development of interventions and technologies that target balance control is needed. The purpose of this study was to identify and explore the priorities for balance-focused interventions and technologies from the perspectives of end-users to assist with the design of an intervention that combines functional electrical stimulation (FES) with visual feedback training for standing balance.

**Methods:**

Two individuals with SCI/D, one individual with stroke, two physical therapists (PT) and one hospital administrator were recruited. Participants attended three focus group meetings that followed a participatory co-design approach. A semi-structured interview guide, developed from the FAME (Feasibility, Appropriateness, Meaningfulness, Effectiveness, Economic Evidence) framework, was used to lead the discussion, querying participants’ experiences with balance deficits and interventions, and FES. Meetings were audio-recorded and transcribed verbatim. An iterative and reflexive inductive thematic analysis was applied to the transcripts by three researchers.

**Results:**

Four themes were identified: (1) Balance is meaningful for daily life and rehabilitation. Participants acknowledged various factors influencing balance control and how balance deficits interfered with participation in activities. End-users stressed the importance of continuing to work on one’s balance after discharge from hospital-based rehabilitation. (2) Desired characteristics of balance interventions. Participants explained that balance interventions should be tailored to an individual’s unique needs and goals, relevant to their lives, balance their safety and risk, and be engaging. (3) Prior experiences with FES to inform future therapeutic use. Participants with stroke or SCI/D described initial apprehension with FES, but experienced numerous benefits that motivated them to continue with FES. Challenges with FES were mentioned, including wires, cost, and time of set up. (4) Potential role of FES in balance interventions. Participants felt that FES would complement balance interventions; however, they had not experienced this combination of therapies previously.

**Conclusions:**

End-users described how their experiences with balance deficits, rehabilitation, and FES informed their priorities for balance interventions. The findings inform the design and implementation of future balance interventions for individuals with SCI/D or stroke, including an intervention involving FES and visual feedback training.

## Introduction

Falling is a health care crisis in many parts of the world due to its prevalence and effects. The global, age-standardized prevalence of falls is 5,186 per 100,000 people [1]. Falls have a significant effect on health care spending, with 8.7 billion Canadian dollars and 49.5 billion American dollars spent on falls-related injuries every year in Canada and the United States, respectively [2, 3]. Falls may result in serious injuries, with the most common including fractures, concussions, and post-fall syndrome; characterized by a fear of falling that results in avoidance of activities that the person could physically perform [1, 4]. Post-fall syndrome is common, with 47–63% of people with spinal cord injuries or diseases (SCI/D) and 49% of people with stroke reporting a concern about falling that limits activity and participation [5–7], and it results in a loss of independence, reduced balance confidence, depression, and immobilization [8, 9]. People at the highest risk of falls are individuals living with neurological injury or disease. For example, approximately three quarters of people with SCI/D and stroke were found to fall at least once per year [1, 10].

Balance control, or the ability to maintain or restore one’s centre of mass (COM) within one’s base of support (BOS), is a modifiable risk factor for falls, and is independently associated with fall risk [11]. When a person’s COM moves outside of their BOS, muscle activation and coordination help to reposition the centre of mass [12]. Impairments in balance control, such as reduced functional stability limits, increased postural sway in standing and reduced or absent reactive responses, are common for individuals with SCI/D or stroke due to somatosensory and motor deficits from the neurological damage [13, 14]. People with stroke may have the added complexity of an impaired perception of verticality, which may impact their postural control [13]. Deficits in balance control affect people with SCI/D and stroke psychologically as well, by having an impact on their capacity for independent living, physical activity levels, perceptions of disability, and overall quality of life [15–18].

Although balance deficits are prevalent in these populations, only a few hours of physical therapy time are spent on balance training during inpatient SCI/D rehabilitation [19]. Similarly, during inpatient stroke rehabilitation, 12% of physical therapy time is spent on standing interventions, with only approximately half of these sessions including a balance component [20]. Due to the time constraints of clinical practice, effective and efficient interventions targeting balance control are needed and hence, their development is a current research priority.

One intervention that may be combined with the movement-based activities that are common in physical therapy is functional electrical stimulation (FES). To date, there are few reports of the incorporation of FES into balance exercises for those living with SCI/D or stroke [21–23]. Recently, a balance intervention combining functional electrical stimulation (FES) with game-based visual feedback balance training (VFBT) was developed for people living with SCI/D [24]. This intervention included a closed-loop controller for the FES system, which mimicked the physiological control system [25–27]. Participants stood on a force plate with their centre of pressure (COP) presented on a monitor. As the participant moved their COP in response to a game, FES was delivered to the plantar flexor and dorsiflexor muscles, with the stimulation intensity regulated in a closed-loop manner based on COP position [28]. This prototype FES + VFBT system was tested on five individuals with incomplete SCI/D, and clinically significant improvements in balance ability were found in four participants after only 12 sessions [24]. Although this system has promise as a new balance intervention, the system likely has low clinical utility given the high cost and low portability of the equipment (e.g. force plate and stimulators), and the specialist training required to operate the equipment [29]. Thus, there is a need to redesign the FES + VFBT system to ensure the intervention’s usability, feasibility, and relevance in current health care environments. Consultations with the end-users of interventions during the early phases of technology and intervention development may influence the design and increase the likelihood of clinical translation [30]. Hence, this study’s purpose was to explore end-user views and priorities for balance and FES interventions, with the intent of directing the development of a clinically feasible FES + VFBT system and other future balance interventions.

## Methods

We completed an exploratory descriptive qualitative study that followed the principles of participatory design, which is a collaborative and iterative approach where end-users of a technology or intervention are involved as informants and co-designers throughout its development [31–33]. Spinuzzi (2005) describes three sequential phases of participatory design: exploration, discovery and prototyping. This study reports on the exploration phase, the methods for which include the research team and end-users sharing their experiences, values and goals related to balance deficits, balance training and FES, and then using this information to reach consensus on desired characteristics and outcomes of balance interventions, including the FES + VFBT system. Four types of end-users of balance interventions collaborated with the research team on this study: individuals with incomplete SCI/D, individuals with stroke, physical therapists (PT) and hospital administrators. Hospital administrators were considered end-users since they typically make decisions regarding equipment purchases and allocation of resources (e.g., staff, time, funds). All study activities were performed in accordance with the principles of the Declaration of Helsinki and ethical approval for this study was obtained from the Research Ethics Board of the University Health Network, Toronto, Canada. All participants provided written, informed consent prior to beginning study activities.

### Study participants

Convenience sampling of end-users was conducted at three rehabilitation hospitals within southern Ontario from February – April 2021. Recruitment notices were sent to staff via email and word of mouth. Anticipated targeted sampling generated “a new and richly textured understanding of experience” to achieve non-redundant saturation of the experiences [34]. The targeted sampling included two individuals with motor incomplete SCI/D (American Spinal Injury Association Impairment Scale C or D), two individuals with stroke, two PT and two hospital administrators, all with knowledge of balance interventions and FES for individuals with neurological impairments. Thus, the targeted sample size was eight participants, which when combined with the research team members would result in the recommended focus group size (i.e., 6–12 participants) [35]. All participants with a SCI/D or stroke also met the following inclusion criteria: (1) were in the chronic phase of recovery (i.e., > one year post-incident), (2) were able to stand independently for one minute, and (3) reported deficits in their balance (i.e., answered yes when asked “Do you feel your balance is poor such that you are at an increased risk of falls?”). These inclusion criteria ensured the participants’ level of function aligned with that targeted by FES + VFBT. At least one PT must have had experience working with individuals with motor incomplete SCI/D. Similarly, at least one PT must have had experience working with individuals with stroke. The hospital administrators must have worked within a neurological rehabilitation unit.

### Data collection

Three focus group meetings were conducted over a web-based platform (Microsoft Teams) and audio-recorded. Each meeting lasted 50–53 min. The first and second meetings occurred one week apart, while the second and third meetings occurred one month apart. In addition to the study participants (i.e. end-users), the following members of the research team (RT) attended the meetings and contributed to the discussions: RT1 (Scientist and PT with expertise in SCI/D rehabilitation and qualitative research), RT2 (Scientist and lead developer of the FES + VFBT system), RT3 (Scientist and PT with expertise in balance interventions), RT4 (Scientist with expertise in implementation science), RT5 (PhD Candidate and engineer who contributed to the development of the FES + VFBT system). Two additional research team members attended the meetings as silent observers to maintain a reflective journal, which detailed participants’ contributions to discussions and non-verbal cues (e.g., facial expressions) deemed relevant to the context of the discussion. Reflective journaling strengthened the rigour and transparency of the study [36].

RT1 led the meetings, using a semi-structured interview guide to direct discussion. The FAME (Feasibility, Appropriateness, Meaningfulness, Effectiveness, Economic Evidence) framework was used to develop the interview guide [30]. This framework can be used to guide the development and testing of rehabilitation technologies and interventions [30]. In brief, the FAME framework encourages researchers and developers to consider the following elements:

- Feasibility: is the intervention practical and practicable?
- Appropriateness: Does the intervention align with current scientific literature, needs of end-users, and current health care contexts and goals?
- Meaningfulness: Do the intervention and potential outcomes matter to the target population?
- Efficacy or Effectiveness: Does the intervention produce the intended effect?
- Economic Evidence: Does the economic evidence support implementation of the intervention?

Questions explored the participants’ experiences and perceptions on balance deficits, balance interventions and FES. At the second meeting, the research team provided an overview of the FES + VFBT system as an example of how FES could be incorporated into balance training [24]. See Table 1 for sample interview questions.

**Table 1:** Sample Interview Questions

Sample Interview Questions	Explanation of FAME Elements Targeted
1. How would you describe your/your patients’ balance?	A: Describes needs of clinician and person with balance deficits M: Considers importance of balance control
2. Can you describe your experience with balance training? [Probes for individuals with SCI/D or stroke: In what settings? What were your goals? What did the balance training involve?] [Probes for PT/hospital administrator: For what clinical populations? In what settings? What interventions and/or technologies?]	F: Explores clinical utility and practicality of balance training A: Describes past populations, settings and resources used for balance training M: Explores meaningfulness with past balance training E: Explores effect of balance training
3. Did you experience any challenges to participating in/delivering balance training? [Probes for individuals with SCI/D or stroke: physical challenges, cost, logistical challenges (e.g., travel to rehabilitation centre), difficulty accessing balance interventions in community, inadequate training environment or expertise of trainers, etc.] [Probes for PT/hospital administrator: cost of equipment, logistical challenges (e.g., training of staff), inadequate space or assistants for balance interventions, etc.]	F: Explores practical challenges with balance training A: Considers challenges of meeting the needs of all end-users EE: Describes economic factors that may have influenced balance training
4. Do you think there is a role for FES in balance training? Why or why not? Please describe that role.	A: Explores the appropriateness of this combined intervention in real-world settings M: Explores importance of combined intervention

Sample questions from the semi-structured interview guide used during the three focus group meetings (left column) along with the FAME elements that each question addresses (right column). SCI/D = spinal cord injury or disease; PT = physical therapists; FES = functional electrical stimulation; F = feasibility; A = appropriateness; M = meaningfulness; E = efficacy/effectiveness; EE = economic evidence

### Data analysis

Audio-recordings of the meetings were transcribed verbatim by a member of the research team (KB or KC). A reflexive thematic analysis was applied to the transcribed data [37]. First, each meeting transcript was read multiple times independently by three researchers (NLB, KB, KEM) and meaningful quotes were then highlighted, and marginal notes added. Highlighted quotes, deemed to have an overarching meaning, were assigned as codes, and codes with similar meanings were brought together as a subtheme [37]. Afterwards, the three researchers discussed the codes with their associated meaningful quotes and reflexively discussed subthemes and overarching themes to interpret the underlying meaning of the codes [37]. The primary reviewer (NLB) was a PhD student with a background in physical therapy who had two years of experience in SCI/D and stroke rehabilitation and engaged in both qualitative and quantitative research. The secondary reviewer (KB) was an undergraduate biomedical engineering student. The tertiary reviewer (KEM) was a PT with 18 years of SCI and stroke rehabilitation experience, and well-versed in qualitative and quantitative research. Subsequently, a thoughtful clinician test was performed by a PT with qualitative research experience (HJR). A thoughtful clinician test is performed through having an expert in the phenomenon of interest confirm that the qualitative claims are plausible and illuminate patterns within the transcriptions previously not explored [37].

## Results

Six end-users (three men, three women) participated in the study: two individuals with chronic SCI/D, one individual with chronic stroke, two PT, and one hospital administrator with an occupational therapy background. Due to the COVID-19 pandemic, participant recruitment was more challenging than anticipated. A second hospital administrator had consented to the study but withdrew prior to the first focus group meeting due to pandemic-related work demands.

Overall, four themes were identified that summarized end-users’ perceptions of their or their patients’ balance, balance interventions and FES. The four themes include (1) Balance is meaningful for daily life and rehabilitation, (2) Desired characteristics of balance interventions, (3) Prior experiences with FES to inform future therapeutic use, and (4) Potential role of FES in balance interventions. Each theme was further divided into 2–4 subthemes, the titles for which were derived from representative quotes from participants (see Table 2). See Tables 3, 4, 5 and 6 for supporting quotes, which are referenced in the text as Q1, Q2, etc.

**Table 2:** Themes and Sub-themes

Themes	Sub-themes
1. Balance is meaningful for daily life and rehabilitation	1a. “Without balance you cannot do anything”
	1b. “I’m still working on my balance”
	1c. “Balance is very multi-faceted”
	1d. “A lot of individual variation”
2. Desired characteristics of balance interventions	2a. “The importance of tailoring the program”
	2b. “The importance of the fun and engagement factor”
	2c. “The balance between risk and safety”
	2d. “Relevant to their lives”
3. Prior experiences with FES to inform future therapeutic use	3a. What individuals with SCI/D or stroke liked about FES
	3b. What therapists liked about FES
	3c. “Challenges we have experienced”
4. Potential role of FES in balance interventions	4a. “They would complement each other”
	4b. Exploring how to incorporate FES into balance training

FES = functional electrical stimulation, SC/D – spinal cord injury or disease

**Table 3:** Theme 1: Balance is meaningful for daily life and rehabilitation

Subtheme	Quotes
1a) “Without balance you cannot do anything” (SCI/D2)	Q1: “… I work for (organization). I have to have a very strong balance in a lot of situations and so this is also the first point of view, balancing is important.” (STR1) Q2: “The one…transition that might not [have been] mentioned - the transition to driving a car…because having the ability to drive is certainly a huge sense of freedom.” (SCI/D2) Q3: “The balance concerns and the balance deficits are the real challenges for our patients and then they really hinder them from being able to, you know move on or be able to do the functions that they need to in order to be, you know, independent or get that level of function that they need to sometimes live independently. So, I’m seeing that sometimes that can be a barrier for us in terms of being able to move patients to their next locations, but also for the patients themselves in feeling, you know, that they aren’t able to have that level of independence for some of the things they were able to manage before quite independently.” (HA1)
1b) “I’m still working on my balance” (SCI/D1)	Q4: “I think you have to develop some kind of exercise program and I think you have to stick with it because I know the difference if I’m not really watching what I’m doing. I’m not doing my exercises every week, all of a sudden, you say, “Oh, that’s a little tough.” (SCI/D2) Q5: “This is my number one goal, yeah, balance.” (SCI/D1) Q6: “And, I remember [PT] really pushed me to do it and then I mean I have to thank [PT] because I am walking now, but of course with two canes and walker, and I’m still worried about my balance.” (SCI/D1) Q7: “I walk every day, but sometimes I don’t trust my balance.” (SCI/D1) Q8:“Everyone will have some kind of, may have some kind of balance deficit after their injuries, whether stroke or spinal cord” (PT1)
1c) “Balance is very multi-faceted” (PT2)	Q9: “… in terms of describing balance with patient populations that I’ve worked with, I would say that it can be quite diverse, right? It can be…different balance deficits sort of in terms of range of degree of impairment, or severe or less severe. And, there could be lots of different reasons for why someone is having the balance deficits.” (RT3) Q10: “I also am quite fascinated with the role that the upper extremity might play and how do we, you know challenge the balance, but also involve maybe the more affected limb, the upper limb.” (PT2) Q11: “…sometimes if you have a quick [reflex] then spasticity kicks in.” (STR1)
1d) “A lot of individual variation” (RT3)	Q12: “… there could be lots of different reasons for why someone is having the balance deficits that they are, and that they might be different person to person. And they may be affecting individuals’ goals very differently as well.” (RT3) Q13: “… we have different levels of balance deficits that we have to deal with because of the ways that or the how patients present to us.” (HA1)

### Theme 1: Balance is meaningful for daily life and rehabilitation

Participants acknowledged the variety of factors influencing balance control. Participants stressed the importance of continuing to work on improving one’s balance after discharge from hospital-based rehabilitation and described how balance deficits interfere with their participation in some daily and therapeutic activities. The theme was subcategorized into the following subthemes: 1a) “Without balance you cannot do anything,” 1b) “I’m still working on my balance,” 1c) “Balance is very multi-faceted,” and 1d) “A lot of individual variation.” See Table 3 for supporting quotes.

#### Subtheme 1a. “Without balance you cannot do anything”

Participants described how balance control was important for independence and participation. Balance was seen as essential for their everyday activities, including the demands of their workplace (Q1). Participants also believed that with balance comes a sense of freedom. In this, they are describing the independence one achieves, such as driving a car (Q2), when you have effective balance control. There was a continued sense of hope for their balance to improve. The hospital administrator added that a lack of balance control could prevent optimal participation in rehabilitation and moving people through the continuum of care (Q3). Here, the hospital administrator indicated how balance and its impact on functional independence can be the cause of longer hospital stays and being unable to move through the hospital system.

#### Subtheme 1b. “I’m still working on my balance”

The importance of continuing to work on improving one’s balance after discharge from hospital-based rehabilitation was stressed by all participants with lived experience. SCI/D2 stated, “My balance has somewhat regressed a little bit because of the COVID I think. I used to exercise regularly at the gym”, and also said, “I agree that it’s an everyday type of thing, and you have to stay on top of that for sure.” Participants with lived experience acknowledged that maintaining balance is challenging, needs to be maintained throughout their lives (Q4), and is one of their top priorities and goals (Q5). Another consideration for participants with lived experience was that they continued to walk with gait aids and worried about their balance (Q6). All participants suggested that anyone who sustains a SCI/D or has a stroke will have ongoing balance impairments (Q7, Q8).

#### Subtheme 1c. “Balance is very multi-faceted”

Participants acknowledged the variety of factors influencing balance control, including severity of neurological damage (Q9), upper limb involvement (Q10), and spasticity (Q11). As summed up by one of the therapists: “… we think about balance and it is quite dynamic, no pun intended.” (PT2) Moreover, participants discussed how each person experiences a different combination of the factors impacting balance control. As PT2 stated: “is it the visual system that’s contributing? Is it the sensory system that’s part of it?… balance is very multi-faceted.”

#### Subtheme 1d. “A lot of individual variation”

Balance deficits were seen to impact individuals’ ability to mobilize in the community as well as to successfully complete their activities of daily living. As stated by researcher and therapist RT3: “… a balance deficit might be impacting on a person’s ability to do activities of daily living or mobility … so there can be a lot of individual variation depending on the individual”. Participants agreed with this statement and also suggested that there was considerable individual variation in which daily activities were impacted and hence, what the wants of the person with lived experience are (Q12). Each patient has their unique level of function, type of balance deficit, and personal goals, which will impact their treatment differently (Q13).

### Theme 2: Desired characteristics of balance interventions

Participants described the principles and concepts that would be important to include in balance interventions, which are outlined in the following subthemes: 2a) “The importance of tailoring the program,” 2b) “The importance of the fun and engagement factor,” 2c) “The balance between risk and safety,” and 2d) “Relevant to their lives.” See Table 4 for supporting quotes.

**Table 4:** Theme 2: Desired characteristics of balance interventions

Subtheme	Quotes
2a) “The importance of tailoring the program” (PT2)	Q14: “… so it does require some thought about what’s right for that person and what are some of the underlying issues that person is having and how best to approach assessment and treatment.” (RT3) Q15: “… understanding the person’s goals, truly meeting them where they’re at, and then constantly thinking of, you know, ways to challenge them and progress.” (PT2) Q16: “… and just doing your assessment and seeing where the limitations are coming from and working towards that.” (PT1)
2b) “The importance of the fun and engagement factor” (PT2)	Q17: “I enjoyed that a lot. I enjoyed the [balance intervention] because it was good for me.” (SCI/D2) Q18: “[Balance intervention] was really fun, yeah.” (SCI/D1) Q19: “… is the importance of the fun and engagement factor. …what can people find engaging and interesting or somewhat fun? Because yeah, to stick with the same program, it can become, you know boring, and so we want to try and find ways that…maintain that engagement, but while still targeting what needs to be targeted.” (PT2)
2c) “The balance between risk and safety” (RT3)	Q20: “… when you think about balance, you have risk and safety… Sometimes I think safety number one, so I don’t worry about my balance, but sometimes I have to risk it.” (SCI/D1) Q21: “I agree that, you know, there’s always risk doing what you’re doing, but I think the risk is necessary…you’ll have analyzed what the risk is going to be. You try to minimize that of course, but then you accomplish something and … you get a bit of confidence.” (SCI/D2) Q22: “We want to really…give people opportunities to move and improve, so there is often this …. safety versus, you know, allowing some risk to improve balance.” (RT3) Q23: “Some patients may be willing to take more risk and so that kind of balancing is an issue…might be difficult for the therapist sometimes…so as I said, consider safety first.” (STR1) Q24: “And, so you retain a level and then you go, “Okay, we’re going to try this.” Again, there’s a risk, but you analyzed it, you worked on it, you try to accomplish it safely, you get more confidence.” (SCI/D2)
2d) “Relevant to their lives” (HA1)	Q25: “…anything that people could find really relevant to their lives or like relevant to a task that they needed to complete or that they could relate to, I felt really helped with people feeling like how that would relate to their function at home or their goals…” (HA1) Q26: “We did a lot of things [in balance intervention] that might not have been, I couldn’t identify as, relevant to my everyday life, but I could identify as being beneficial to my balance, which when I came home, I certainly noticed a difference with reaching up or range of motion sideways. What I particularly enjoyed down there was the working on the uneven surfaces because that certainly challenges your core, challenges your balance.” (SCI/D2) Q27: “…different patients that have come to us and said, “Oh, we want to like, kayak or canoe” or something on water. So I guess one part of challenging balance training for that would be like simulating the environment… So, we’ve tried like sitting on Bosu balls… I guess just like always simulating the environment to really truly make sure they would be safe when they’re trying it for the first time. Because we won’t be in Florida with them. ” (PT1)

#### Subtheme 2a. “The importance of tailoring the program”

The content of balance interventions should be tailored to the individual’s balance deficits (Q14) and therapy goals (Q15). PT2 emphasized “the importance of tailoring the balance program to meet where the person is and where their need, their unique need is and then building in a progressive component within that.” Therapists explained the value of completing an assessment to identify an individual’s limitations in their balance control, which subsequently directs the activities targeted in a balance intervention (Q16).

#### Subtheme 2b. “The importance of the fun and engagement factor”

Another common principle discussed was how important it is for individuals to be engaged throughout a balance intervention. The hospital administrator (HA1) stated: “Anything that you can do to continue to change the task, or modify, or anything you can do to make it more entertaining to participate in, is helpful.” This view was shared by all participants; having fun kept them engaged in the balance training (Q17, Q18). Participants stated that balance interventions needed to be fun, engaging, challenging, and accomplishing something (Q19).

#### Subtheme 2c. “The balance between risk and safety”

The need to balance risk and safety during therapy and everyday life (Q20) was frequently mentioned by the participants with SCI/D and stroke. They wanted to improve their balance but understood that there would be an element of risk to it (Q21). Overall, participants agreed that a certain amount of risk is acceptable when participating in a balance intervention so that one can improve their balance control and achieve their goals (Q22), but finding the equilibrium between risk and safety can be tricky (Q23). A suggestion made by RT3 was to “… give people opportunities to move and improve…,” and enhance safety as much as possible. Although balance training is necessary, safety should always be the main priority. Everyone has a different comfort level with risk, as stated by STR1, “one of the things is that the patient has to feel comfortable and also needs to be challenged.” There is a need to analyze what the risk is, minimize it, and then participate in a balance intervention to improve confidence in movement and balance performance (Q24).

#### Subtheme 2d. “Relevant to their lives”

All participants stressed the importance of creating balance interventions that had relevance to the lives and goals of individuals living with neurological injury or disease. The hospital administrator (HA1) said: “so I think anything that you can find, that really add[s] some real relevance to the patient, so that they can relate it to their home lives.” Practicing relevant tasks was perceived to help the individual relate to completing the task outside of the therapeutic environment (Q25). One participant with SCI/D added that it was important to see how a balance intervention was relevant to one’s balance deficits, and that not all balance interventions needed to clearly link to functional activities (Q26). Therapists acknowledged the challenge of creating balance exercises that simulated some real-world tasks, and discussed the need to be creative in the delivery of balance interventions (Q27). As stated by PT2: “… meeting what their interests are and what their goals are and then creating an environment using the built environment, using the tools you have access to, to sort of recreate or simulate that as close as possible.”

### Theme 3: Prior experiences with FES to inform future therapeutic use

All participants had prior experience with FES. Therapists and the hospital administrator discussed using multi-channel systems, such as the MyndMove® and Xcite®, in addition to hand-held one- and two-channel devices. Participants suggested that FES was applicable across the continuum of care (i.e., inpatient, outpatient, and community rehabilitation) due to its versatility. This theme was categorized into the following subthemes: 3a) What individuals with SCI/D or stroke liked about FES, 3b) What therapists liked about FES, and 3c) Challenges of FES. See Table 5 for supporting quotes.

**Table 5:** Theme 3: Prior experiences with FES to inform future therapeutic use

Subtheme	Quotes
3a) What individuals with SCI/D or stroke like about FES	Q28: “But, I trusted the team. I wish I could do it again, I mean it was really helpful, really, really helpful.” (SCI/D1) Q29: “… the FES I did for a while, I could see the improvement in motion … I could sense sensations in muscles becoming activated and I could tell that I was getting better.” (SCI/D2) Q30: “I noticed an increase in range of motion, especially leaning forward, leaning to the side, basically it worked well for me. I felt it was really beneficial once I left the program for sure, because it had awakened some muscles.” (SCI/D2)
3b) What therapists liked about FES	Q31: “…lots of the things that we love about it is the ability to truly target multiple muscle groups and engage and produce movement most folks have not experienced for a really long time.” (PT2) Q32: “And, then again as a therapist, sort of the creativity that comes into that is how do you shape the environment to build in a meaningful task for that person? And you’re doing that either in sitting or standing, again just adding the creativity piece into it, always thinking about that in the back of your mind…you’re the technical application of the FES.” (PT2)
3c) “Challenges we have experienced” (HA1)	Q33: “…because of the cables…wireless may be better.” (STR1) Q34: “So, I think you’re sort of restricted to some extent of what [you] can do with the length of the cable that you have. And the way that sometimes we’ve worked around it is by placing the device on a movable surface, or you know moving our actual stim device off of, you know, like a wheelie.” (PT2) Q35: “One challenge with that I think though is how expensive it is, essentially. So, you can train someone with it for, I don’t know an hour, an hour and a half, but then they have nothing for them to do for the rest of the week…” (PT1) Q36: “So, how do we have a program for them to also work on that at home and not just in therapy?” (PT1)

#### Subtheme 3a. What individuals with SCI/D or stroke liked about FES

Participants with SCI/D or stroke described initial apprehension about using FES; as stated by SCI/D1: “it was very challenging for me, and I was scared at the beginning.“ However, their apprehension was alleviated by working with their therapy team (Q28) and experiencing benefits that motivated them to continue with FES. Participants with SCI/D or stroke discussed what they liked about FES from their experiences. One participant, SCI/D2, reported experiencing numerous benefits: “I found FES to be very beneficial - improved quality of life, was able to achieve actual physical motion. FES helped improve confidence in new movements.“ Participant SCI/D2 also felt increased sensations in lower limb muscle (Q29), increased range of motion (Q30), and increased dynamic sitting balance. Subjectively, FES woke their muscles, with SCI/D2 stating, “My right side, even still today, is progressing a little bit, and I think that’s kind of got woken up…”.

#### Subtheme 3b. What therapists liked about FES

Therapists indicated that they liked FES because it targets specific muscle groups and produces movement that would otherwise not occur (Q31). As explained by PT2: “It’s not just, you know, a crude extension/flexion of the wrist. It’s actually a functional movement of reaching and grasping and releasing, and having the electrical stim facilitate that, that has been really nice.” Therapists discussed liking the creativity required of FES applications; creativity to shape an environment and build a meaningful task (Q32).

#### Subtheme 3c. “Challenges we have experienced.”

Although there were many positives of using FES, participants discussed the challenges with FES as well. The participants with SCI/D or stroke commented on the cables being a nuisance (Q33). Something noticed by SCI/D2 was: “it did take the guys a long time to set up sometimes.” Therapists and the Hospital Administrator also commented that the length of the cables could impact the activity practiced in therapy (Q34). When discussing the MyndMove®, PT2 stated “There’s a lot of cables. It’s not a small compact unit.” Therapists discussed the cost of FES (Q35) and the inability to translate the therapy to a home environment (Q36) as additional challenges. The hospital administrator (HA1) summarized the numerous challenges:

I would say the biggest challenges [were]…time, you know getting people down to set them up on the equipment and then having the time to continue that you make sure that you’re, you know using it as it’s intended to be so that intensity, that frequency. And, then of course some of these things are expensive, so just being able to purchase some of the more like sophisticated equipment. I’d say those are kind of the challenges we have experienced.

### Theme 4: Potential role of FES in balance interventions

When asked about the potential role of FES in balance interventions, participants felt that FES would complement balance interventions; however, they had not experienced this combination of therapies in their rehabilitation or clinical practice. This theme was organized into the following subthemes: 4a) “They would complement each other” and 4b) Exploring how to incorporate FES into balance training. See Table 6 for supporting quotes.

#### Subtheme 4a. “They would complement each other”

Participants were in favor of incorporating FES into balance interventions. As stated by SCI/D2: “When you put both of those together, it would be great, um, because I think they would complement each other.” The participant with stroke (STR1) commented that the possibility of FES being used to help one’s balance control was “very exciting”. The participating therapists agreed with this statement (Q37). The hospital administrator added that the scientific evidence supporting FES justified its use in balance interventions (Q38). Participants stated that FES combined with exercises that challenge one’s balance control would be engaging and a useful addition to functional mobility interventions. Also, electrical stimulation could facilitate muscle contractions in response to a change in the base of support or a loss of balance. Since balance deficits are a barrier to progression in rehabilitation, participants noted that FES may assist individuals in attaining a higher level of function during therapy. Therapists had a sense of hope and were excited by the many possibilities and applications of FES paired with balance interventions (Q39).

#### Subtheme 4b. Exploring how to incorporate FES into balance training

Participants discussed various ways that FES could be incorporated into balance interventions in the future (Q40). One suggestion was that an FES application for gait may be used to assist with reactive balance responses when walking (Q41). It could also be used for postural control by activating the trunk (Q42). Participants with SCI/D and stroke agreed that “FES procedures in the future should create better balance and posture” (SCI/D2).

**Table 6:** Theme 4: Potential role of FES in balance interventions

Subtheme	Quotes
4a) “They would complement each other” (SCI/D2)	Q37: “… but I’m starting to get excited when I listen to all of this because I can think of a lot of ways that we could start to use [FES] in balance training.” (RT3) Q38: “… there’s so much evidence for functional electrical stimulation around, you know, any neurological recovery, right? So like spinal cord, stroke. And the evidence is really promising, right? And we’re seeing it come out in a lot of best practice guidelines… So to me it’s almost kind of like a no-brainer, you know?” (HA1) Q39: “I mean I can see some exciting pairings of what therapists typically do for balance training and the role of FES… So, I mean we’re trying to use tasks to elicit some of those muscles, but then to pair those tasks with FES would, I could see some really neat um neat things happening.” (L1)
4b) Exploring how to incorporate FES into balance training	Q40: “Like while they were either in standing or in sitting and again having them do some of those uh reaching tasks outside of there…I think just what everybody else is saying is we do…a little bit of exploring …” (HA1) Q41: “… we typically would use [FES] as an adjunct to whether it’s gait training and activation and then we would challenge balance separately, so this is really exciting. I think one of the things I would use is the Bioness and then, again it’s more within the um gait training components, but sometimes there were moments when there would be a reactive balance, where you would lose control and then you’d have the Bioness on …” (PT2) Q42: “The other point that…was interesting was about that postural control because even like with my OT role, I would use the Xcite a lot just to kind of activate the trunk.” (HA1)

## Discussion

The findings in this study have implications for the design of balance interventions for people with SCI and stroke. The first theme, “Balance is meaningful for daily life and rehabilitation,” emphasized how balance deficits impact daily activities and are an ongoing challenge for people with SCI/D or stroke. The second theme, “Desired characteristics of balance interventions,” discussed high priority wants and needs of end-users of balance interventions (i.e., PT, hospital administrators and people living with SCI/D or stroke). The third theme, “Prior experience with FES to inform future therapeutic use,” acknowledged the aspects of FES that are enjoyed by the end-users, but also the challenges end-users have with FES. The fourth theme, “Potential role of FES in balance interventions,” identified this combination of interventions as desired by end-users to improve balance goals. Altogether, the study findings incorporated end-users’ informative experiences to shape recommendations for the design and implementation of balance interventions.

### The importance of balance control to end-users

For the participants of this study, improving balance control following SCI/D or stroke was a rehabilitation priority, as seen in subthemes 1a and 1b. In these subthemes, participants with lived experience discussed the need to constantly work towards improving their balance control because they see it as essential for their independence and participation in daily activities. Previous literature is mixed on the importance of balance control to people with lived experience. For example, a quantitative questionnaire study found that balance was not a priority for people with SCI/D, with only 11% of people with quadriplegia and 16.5% of people with paraplegia ranking balance as an important goal for themselves [38]. However, in this questionnaire, balance was grouped with upper body strength as “upper body/trunk strength and balance,” with no specific category for balance control in general or when standing or walking [38]. Considering prior qualitative research, falls prevention via balance interventions has been identified as a top-priority for both people with SCI/D and stroke, and is the most frequently cited exercise component to include in interventions [39]. A person’s risk of falls causes them to give up on recreational participation [40] and alter, avoid, or depend on others to carry out their activities of daily living [41, 42]. Participants with lived experience also described how their impaired balance impacted their emotional well-being through reduced independence, encounters with inaccessible community environments, and a constant need to focus on safety within everyday tasks [40–43].

Prior research involving clinicians and healthcare administrators suggest these professionals view falls as a significant rehabilitation issue. In a survey study querying clinician perspectives of falls among people with stroke, 74% of respondents reported falls interfering with therapeutic outcomes, and 85% of PT viewed falls as an essential problem within their rehabilitation practice [44]. Prior qualitative studies involving hospital administrators [45] and physical and occupational therapists [46] working in SCI/D rehabilitation highlighted the impact of fall risk on rehabilitation goals, activities and policies. For example, hospitals’ zero falls policies cause therapists to take a precautionary approach to rehabilitation instead of exploring and building insight into patients’ abilities and limits. These findings are akin to those found in this study.

### Recommendations for balance interventions

The study objective was to explore end-user views and priorities for balance and FES interventions to direct the development of a clinically feasible FES + VFBT system. Our findings inform recommendations that may increase the feasibility, applicability and meaningfulness of balance interventions, including the FES + VFBT intervention (Table 7). These recommendations may be relevant to clinicians who create program- and patient-level treatment plans for people living with neurological injury or disease, and/or to researchers and developers who are designing novel balance interventions. For example, the next step of our research is to apply these recommendations to the redesign of the FES + VFBT intervention and subsequently evaluate its acceptability, from the perspectives of patients and clinicians, and its efficacy.

**Table 7:** Recommendations for the design and implementation of balance interventions for individuals with SCI/D or stroke

Themes	Recommendations for Balance Interventions
Balance is meaningful for daily life and rehabilitation	1. Target balance control in the rehabilitation of people with stroke or SCI/D across the continuum of care. 2. Need balance interventions to be available for daily use to maintain and improve balance control.
Desired characteristics of balance interventions	1. Balance interventions should be relevant to the patients’ activities, lives and homes, and to their individual balance deficits. 2. Balance interventions should be fun and engaging. 3. Balance interventions should prioritize safety to increase patient comfort with the intervention, yet also offer a degree of challenge as appropriate for each individual’s risk tolerance.
Prior experiences with FES to inform future therapeutic use	1. The set-up time required for FES interventions should be minimized. 2. Wireless FES interventions are desired by end-users. 3. Inexpensive FES interventions are desired by end-users. 4. Home therapy options for FES should be explored.
Potential role of FES in balance interventions	1. Balance interventions combined with FES should be explored.

Study participants discussed the need to consider safety versus risk for balance interventions. Comfort with risk varies from person to person; however, a certain degree of challenge may be a requisite for effective balance training. The findings of previous studies suggest that balance interventions may be infrequently used because therapists do not feel comfortable administering the interventions, resulting in patients not practicing movements near their limits of stability [46, 47]. Therapists working in SCI/D rehabilitation acknowledged this bias toward safety in practice as being “unfair” to patients and due to hospital policies focused on zero falls [46]. Ways to increase patient safety while performing challenging balance interventions do exist; for example, individuals with SCI/D stated that donning safety harnesses increased their comfort and confidence with balance interventions [24, 48, 49], which may lead to improved participation with the therapy.

End-users desire fun and engaging balance interventions, but what makes an intervention engaging to enhance participation? Burke et al. discuss two main concepts within game design theory to enhance engagement in rehabilitation: meaningful play and challenge [50]. By meaningful play, Burke et al. suggest needing purpose and meaning through direct feedback within the intervention [50]. Challenge is created by making the intervention easier at the beginning for early success and progressively increasing the difficulty to match the user’s ability level [50, 51]. Group-based interventions have also been suggested to increase engagement in rehabilitation and exercise interventions [50, 52]. These suggestions can be utilized to enhance the engagement of balance interventions.

Study participants also expressed an interest in incorporating FES into balance interventions, but did not have prior experience with this combination of interventions. One participant also mentioned they were fearful of trying FES at first, suggesting that strategies to assist in combating apprehension may be of use. There are several prior studies that have incorporated FES into balance interventions in research environments, including the FES + VFBT intervention that our team developed [24, 27, 28, 53] and an electromyogram (EMG)-trigged FES standing balance intervention for people with stroke [21]. The latter device detects muscle activation in the gastrocnemius and tibialis anterior bilaterally through EMG, which subsequently will activate the FES for the same muscle group [21]. FES has also been incorporated into a perturbation-based balance intervention [23] for people living with SCI/D and weight-shifting training in standing for people with stroke [22]. This prior research speaks to the feasibility of incorporating FES into balance interventions, while the results of the current study suggest end-users of balance interventions feel the combination of FES and balance training is appropriate.

One limitation of the current study is the small sample size. The targeted sample size was two participants from each end-user group; however, the COVID-19 pandemic limited the ability of some potential participants to participate. One hospital administrator withdrew from the study prior to the first focus group due to pandemic-related workload demands. We were also unable to recruit a second individual living with a stroke. Another study limitation was that all participants lived or worked in urban areas in one Canadian province. Hence, the study findings and resulting recommendations should be interpreted with caution.

In conclusion, this study explored end-users’ experiences and perceptions of balance deficits, balance interventions and FES. The findings were used to create recommendations for the development of future balance interventions and technologies that are feasible, applicable, and meaningful for the rehabilitation of individuals with SCI/D or stroke.

## Data Availability

The data that support the findings of this study may be available from the corresponding author (KEM) upon reasonable request and with permission of the Research Ethics Board of the University Health Network.

## Abbreviations

COM: Centre of mass
COP: Centre of pressure
EMG: Electromyography
FAME: Feasibility, Appropriateness, Meaningfulness, Effectiveness, Economic Evidence
FES: Functional electrical stimulation
PT: Physical therapist
RT: Research team
SCI/D: Spinal cord injury/disease
VFBT: Visual feedback balance training
